# (3aR*,8bR*)-3a,8b-Dihy­droxy-1-(4-methyl­phen­yl)-2-methyl­sulfanyl-3-nitro-1,8b-di­hydro­indeno­[1,2-*b*]pyrrol-4(3a*H*)-one

**DOI:** 10.1107/S1600536814002712

**Published:** 2014-02-12

**Authors:** R. A. Nagalakshmi, J. Suresh, R. Ranjith Kumar, V. Jeyachandran, P. L. Nilantha Lakshman

**Affiliations:** a Department of Physics, The Madura College, Madurai 625 011, India; b Department of Organic Chemistry, School of Chemistry, Madurai Kamaraj University, Madurai 625 021, India; c Department of Food Science and Technology, University of Ruhuna, Mapalana, Kamburupitiya 81100, Sri Lanka

## Abstract

The asymmetric unit of the title compound, C_19_H_16_N_2_O_5_S, contains four independent mol­ecules (*A*, *B*, *C* and *D*), with two mol­ecules (*B* and *D*) displaying disorder in their methyl­sulfanyl groups [occupancy ratios of 0.797 (11):0.203 (11) and 0.85 (2):0.15 (2)]. The nitro groups are twisted slightly out of the planes of the 2-pyrroline rings to which they are bonded with dihedral angles of 10.17 (1), 8.01 (1), 9.44 (1) and 8.87 (1)° in mol­ecules *A*, *B*, *C* and *D*, respectively. The 2-pyrroline rings are almost orthogonal to the attached tolyl rings, forming dihedral angles of 73.44 (1), 81.21 (1), 88.18 (8) and 73.94 (1)° for mol­ecules *A*, *B*, *C* and *D*, respectively. A weak intra­molecular O—H⋯O inter­action is observed in mol­ecules *B* and *C*. The two hy­droxy groups in each mol­ecule are involved in inter­molecular O—H⋯O hydrogen bonding. In the crystal, mol­ecules are connected *via* O—H⋯O and C—H⋯O hydrogen bonds, forming a complex three-dimensional network.

## Related literature   

For the importance of pyrrolidine and pyrroline derivatives, see: Obniska *et al.* (2002 ▶); Stylianakis *et al.* (2003 ▶); Coldham & Hufton (2005 ▶); Kravchenko *et al.* (2005 ▶); Nair & Suja (2007 ▶); Pandey *et al.* (2006 ▶). For a related structure, see: Nagalakshmi *et al.* (2013 ▶). For ring conformation parameters, see: Cremer & Pople (1975 ▶).
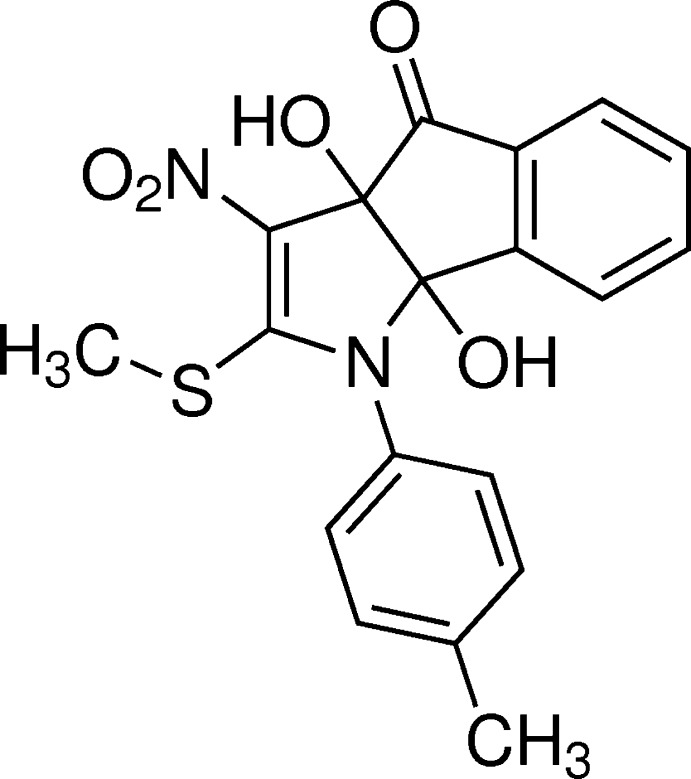


## Experimental   

### 

#### Crystal data  

C_19_H_16_N_2_O_5_S
*M* *_r_* = 384.40
Triclinic, 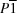

*a* = 10.0830 (5) Å
*b* = 10.2775 (5) Å
*c* = 34.6579 (17) Å
α = 97.540 (3)°
β = 96.028 (3)°
γ = 97.138 (3)°
*V* = 3505.9 (3) Å^3^
*Z* = 8
Mo *K*α radiation
μ = 0.22 mm^−1^
*T* = 293 K
0.21 × 0.19 × 0.18 mm

#### Data collection  

Bruker Kappa APEXII diffractometer
Absorption correction: multi-scan (*SADABS*; Sheldrick, 1996 ▶) *T*_min_ = 0.967, *T*_max_ = 0.974
53951 measured reflections
13050 independent reflections
9856 reflections with *I* > 2σ(*I*)
*R*_int_ = 0.041

#### Refinement  

*R*[*F* ^2^ > 2σ(*F* ^2^)] = 0.069
*wR*(*F* ^2^) = 0.188
*S* = 1.06
13050 reflections
1016 parameters
12 restraints
H-atom parameters constrained
Δρ_max_ = 0.68 e Å^−3^
Δρ_min_ = −0.30 e Å^−3^

### 

Data collection: *APEX2* (Bruker, 2004 ▶); cell refinement: *SAINT* (Bruker, 2004 ▶); data reduction: *SAINT*; program(s) used to solve structure: *SHELXS97* (Sheldrick, 2008 ▶); program(s) used to refine structure: *SHELXL97* (Sheldrick, 2008 ▶); molecular graphics: *PLATON* (Spek, 2009 ▶); software used to prepare material for publication: *SHELXL97*.

## Supplementary Material

Crystal structure: contains datablock(s) global, I. DOI: 10.1107/S1600536814002712/bh2494sup1.cif

Structure factors: contains datablock(s) I. DOI: 10.1107/S1600536814002712/bh2494Isup2.hkl

Supporting information file. DOI: 10.1107/S1600536814002712/bh2494Isup3.cml

CCDC reference: 985344

Additional supporting information:  crystallographic information; 3D view; checkCIF report

## Figures and Tables

**Table 1:** Hydrogen-bond geometry (Å, °)

*D*—H⋯*A*	*D*—H	H⋯*A*	*D*⋯*A*	*D*—H⋯*A*
O3*B*—H3*B*⋯O5*B*	0.82	2.19	2.748 (4)	125
O3*C*—H3*C*⋯O5*C*	0.82	2.17	2.728 (4)	126
O2*A*—H2*A*⋯O3*A* ^i^	0.82	1.97	2.785 (3)	175
O3*A*—H3*A*⋯O3*B* ^ii^	0.82	2.04	2.854 (3)	172
O2*B*—H2*B*⋯O1*A* ^iii^	0.82	2.01	2.787 (3)	157
O2*C*—H2*C*⋯O5*D* ^iv^	0.82	2.57	3.133 (4)	127
O2*C*—H2*C*⋯O1*D* ^iv^	0.82	2.03	2.797 (4)	155
O3*D*—H3*D*⋯O3*C* ^iv^	0.82	2.04	2.857 (3)	175
C10*A*—H10*A*⋯O5*A* ^iii^	0.93	2.54	3.347 (5)	146
C10*C*—H10*C*⋯O5*C* ^v^	0.93	2.50	3.331 (6)	149
C10*D*—H10*D*⋯O5*D* ^v^	0.93	2.59	3.437 (5)	151
C10*B*—H10*B*⋯O5*B* ^ii^	0.93	2.53	3.316 (6)	142
C2—H20⋯O1*C* ^vi^	0.96	2.50	3.446 (7)	170

Symmetry codes: (i) 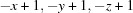; (ii) 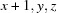; (iii) 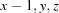; (iv) 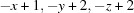; (v) 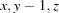; (vi) 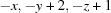.

**Fig. 1.**
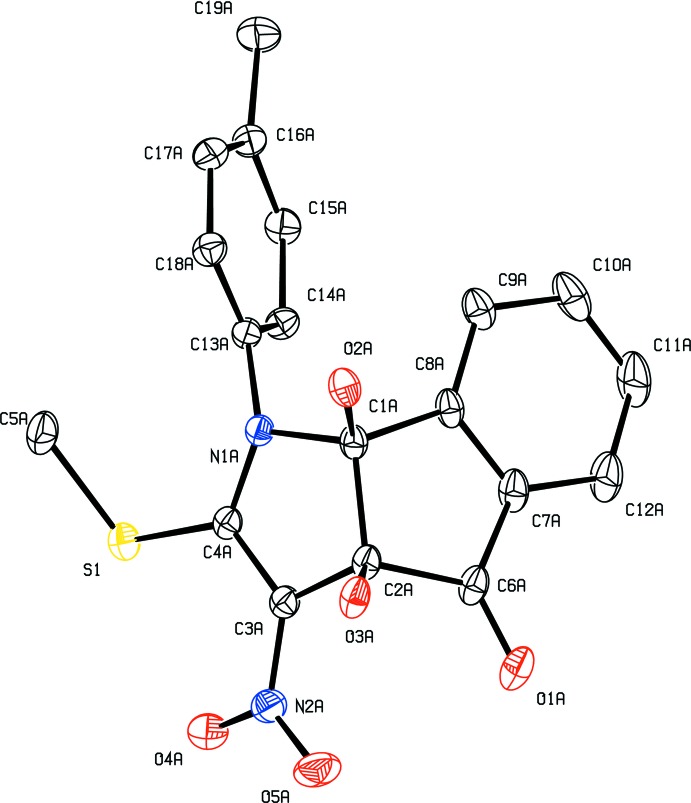
The molecular structure of (I), showing 20% probability displacement ellipsoids and the atom-numbering scheme of molecule A. H atoms are omitted for clarity.

**Fig. 2.**
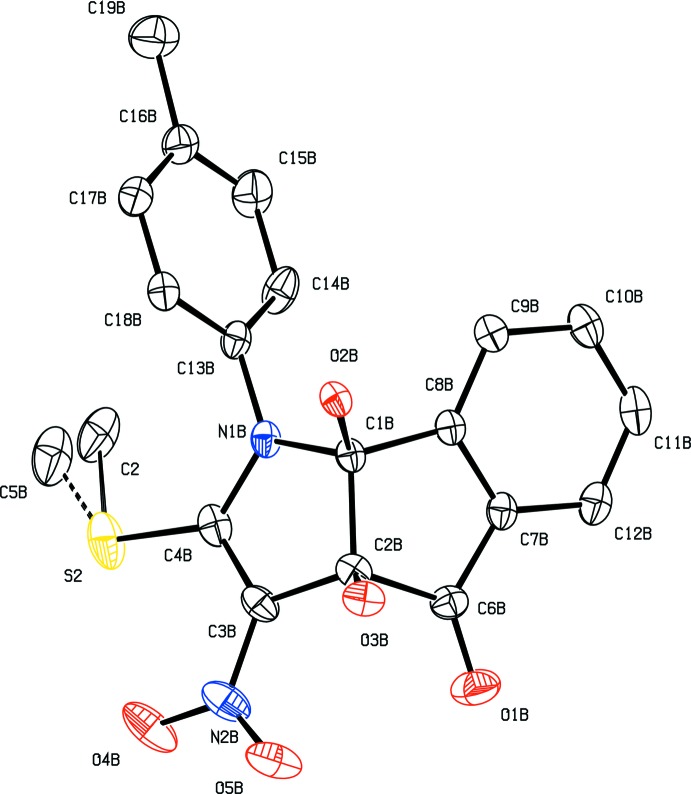
The molecular structure of (I), showing 20% probability displacement ellipsoids and the atom-numbering scheme of molecule B. H atoms are omitted for clarity.

**Fig. 3.**
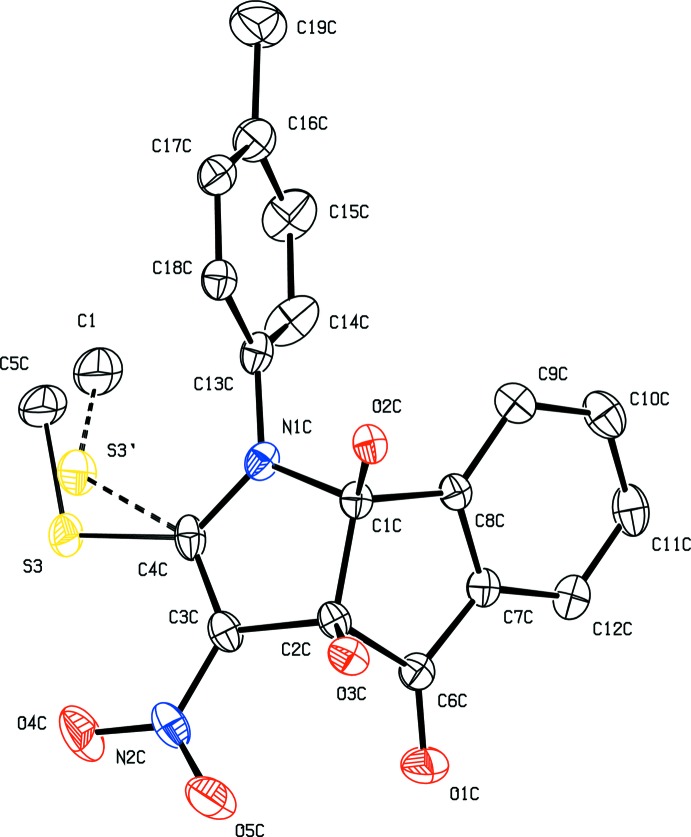
The molecular structure of (I), showing 20% probability displacement ellipsoids and the atom-numbering scheme of molecule C. H atoms are omitted for clarity.

**Fig. 4.**
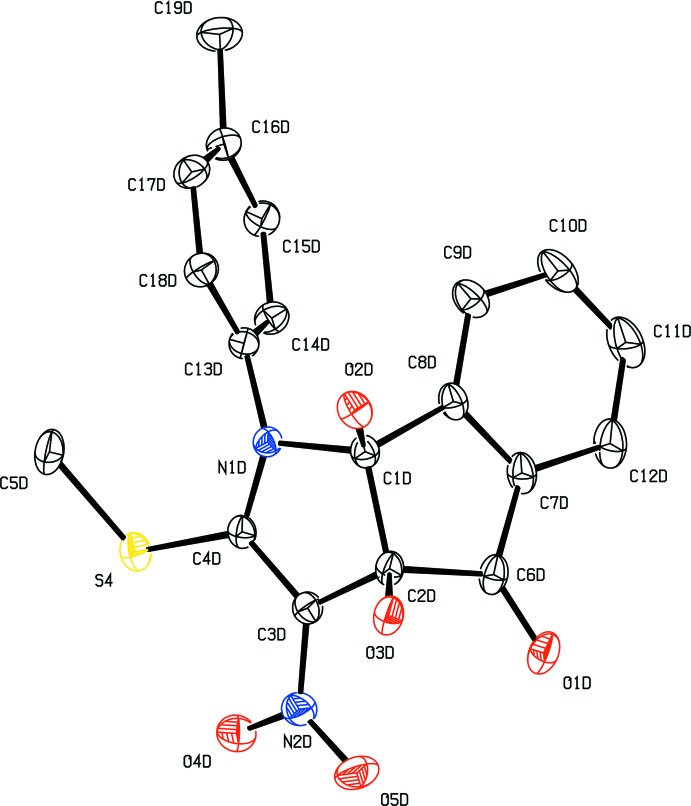
The molecular structure of (I), showing 20% probability displacement ellipsoids and the atom-numbering scheme of molecule D. H atoms are omitted for clarity.

**Fig. 5.**
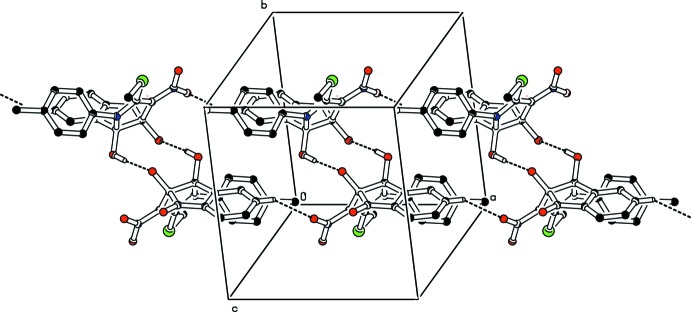
The partial packing diagram showing O—H···O and C—H···O interactions in molecule A.

## supplementary crystallographic information

### 1. Comment

Pyrrolidine-containing compounds form an important class of heterocyclic compounds with wide spread applications to the synthesis of biologically active compounds and natural products (Coldham & Hufton, 2005; Kravchenko *et al.*, 2005; Nair & Suja, 2007; Pandey *et al.*, 2006). Pyrrolidine derivatives possess anti-influenza (Stylianakis *et al.*, 2003) and anti-convulsant (Obniska *et al.*, 2002) activities. The high medicinal values of these compounds, in conjunction with our research interests, prompted us to synthesize and report the X-ray study of the title compound. The same compound with the *p*-tolyl group replaced by a *p*-methoxyphenyl group was previously reported (Nagalakshmi *et al.*, 2013).

The asymmetric unit of the title compound, C_19_H_16_N_2_O_5_S, contains four crystallographically independent molecules *A*, *B*, *C* and *D* (shown in Figs. 1, 2, 3 and 4, respectively), with two molecules (*A* and *C*) exhibiting similar geometries and the other two molecules (*B* and *D*) displaying disorder. The 2-pyrroline rings of molecules *A* and *D* are planar with r.m.s. deviations of 0.0277 (1) and 0.0234 (1) Å; molecules *B* and *C* adopt a slightly twisted conformation for these rings, with puckering parameters *Q* = 0.112 (4) Å, φ_2_ = 231 (2)° and *Q* = 0.080 (4) Å, φ_2_ = 226 (3)°, respectively (Cremer & Pople, 1975). The cyclopentane rings of molecules *A* and *D* are planar. Molecule *B* adopts a twisted cyclopentane conformation, with puckering parameters *Q* = 0.098 (4) Å, φ_2_ = 231 (2)°; molecule *C* adopts an envelope cyclopentane conformation with puckering parameters *Q* = 0.078 (4) Å and φ_2_ = 351 (3)°. The 2-pyrroline rings are almost orthogonal to the attached tolyl rings in all molecules, forming dihedral angles of 73.44 (1), 81.21 (1), 88.18 (8) and 73.94 (1)° for molecules *A*, *B*, *C* and *D*, respectively. The nitro group (N2/O4/O5) is twisted away from the attached 2-pyrroline ring (C1···C4/N1) in all molecules: dihedral angles are 10.17 (1), 8.01 (1), 9.44 (1) and 8.87 (1)° in *A*, *B*, *C* and *D*, respectively. In molecule *B*, the methyl group of the methylsulfanyl functionality is disordered over two positions with occupancies of 0.15 (2):0.85 (2). In molecule *D*, the methylsulfanyl group attached to the 2-pyrroline ring is disordered over two sites, with occupancies of 0.797 (11):0.203 (11).

A weak intra-molecular O—H···O interaction is observed in molecules *B* and *C* (Table 1). The two hydroxy groups in each molecule are involved in inter-molecular O—H···O hydrogen bonding. The C—H···O interactions between molecules form a linear chain along the *b* axis. Intermolecular interactions O2A—H2A···O3A and O2D—H2D···O3D connect the inversion related dimers, forming a ring motif *R*_2_^2^(10) (Fig. 5). The C14D—H14D···O4D inter-molecular interactions form a ring motif *R*_2_^2^(16). These ring motifs are further connected by C—H···O interactions. The O3D—H3D···O3C interaction forms a linear chain along the *b* axis. The C—H···O interactions involving molecules *A* (Fig. 5) and *B* form a linear chain along the *a* axis. The C—H···O interactions between molecules *C* and *D* form a linear chain along the *b* axis. This combination of O—H···O and C—H··· O hydrogen bonds give a complex three-dimensional network for the crystal structure.

### 2. Experimental

A mixture of (*E*)-4-methyl-*N*-[1-(methylthio)-2-nitrovinyl]aniline (1 mmol) with ninhydrin (2,2-dihydroxyindane-1,3-dione, 1 mmol) in presence of glacial AcOH (3–5 drops) was thoroughly ground in a pestle and mortar at room temperature for 2–10 min. The reaction progress was monitored by thin layer chromatography. After completion of the reaction, the reaction mixture was triturated with crushed ice, the resulting solid filtered off and washed with water to afford the pure product. The compound was further recrystallized from ethanol to obtain suitable crystals for X-ray analysis. Yield 96%; m.p. 471–474 K.

### 3. Refinement

H atoms were placed at calculated positions and allowed to ride on their carrier atoms with C—H = 0.93 Å (aromatic CH) or 0.96 Å (methyl CH_3_) and O—H = 0.82 Å. isotropic displacement parameters for H atoms were defined as *U*_iso_(H) = 1.2*U*_eq_(C) for aromatic CH and *U*_iso_(H) = 1.5*U*_eq_(C, O) for CH_3_ and OH groups. Molecules *B* and *C* have disordered parts in their methylsulfanyl fragments. For molecule *B*, the methyl group is disordered over two positions, C2 and C5B, for which occupancies converged to 0.85 (2) and 0.15 (2), respectively. A combination of *SIMU* (similar *U_ij_* parameters for C2 and C5B) and *DELU* (rigid bonds for S2—C5B and S2—C2) restraints were applied (Sheldrick, 2008). In the case of molecule *C* the methylsulfanyl group is disordered over two positions, S3—C5C [occupancy: 0.797 (11)] and S3'—C1 [occupancy: 0.203 (11)]. A *SADI* (same distance) restraint was applied for bonds S3—C5C and S3'—C1.

### Crystal data

**Table:**

C_19_H_16_N_2_O_5_S	*Z* = 8
*M**_r_* = 384.40	*F*(000) = 1600
Triclinic, *P*1	*D*_x_ = 1.457 Mg m^−^^3^
Hall symbol: -P 1	Melting point: 471 K
*a* = 10.0830 (5) Å	Mo *K*α radiation, λ = 0.71073 Å
*b* = 10.2775 (5) Å	Cell parameters from 2000 reflections
*c* = 34.6579 (17) Å	θ = 2–31°
α = 97.540 (3)°	µ = 0.22 mm^−^^1^
β = 96.028 (3)°	*T* = 293 K
γ = 97.138 (3)°	Block, yellow
*V* = 3505.9 (3) Å^3^	0.21 × 0.19 × 0.18 mm

### Data collection

**Table:**

Bruker Kappa APEXII diffractometer	13050 independent reflections
Radiation source: fine-focus sealed tube	9856 reflections with *I* > 2σ(*I*)
Graphite monochromator	*R*_int_ = 0.041
Detector resolution: 0 pixels mm^-1^	θ_max_ = 25.5°, θ_min_ = 0.6°
ω and φ scans	*h* = −12→12
Absorption correction: multi-scan (*SADABS*; Sheldrick, 1996)	*k* = −12→12
*T*_min_ = 0.967, *T*_max_ = 0.974	*l* = −41→41
53951 measured reflections	

### Refinement

**Table:**

Refinement on *F*^2^	Primary atom site location: structure-invariant direct methods
Least-squares matrix: full	Secondary atom site location: difference Fourier map
*R*[*F*^2^ > 2σ(*F*^2^)] = 0.069	Hydrogen site location: inferred from neighbouring sites
*wR*(*F*^2^) = 0.188	H-atom parameters constrained
*S* = 1.06	*w* = 1/[σ^2^(*F*_o_^2^) + (0.0785*P*)^2^ + 4.0405*P*] where *P* = (*F*_o_^2^ + 2*F*_c_^2^)/3
13050 reflections	(Δ/σ)_max_ = 0.001
1016 parameters	Δρ_max_ = 0.68 e Å^−^^3^
12 restraints	Δρ_min_ = −0.30 e Å^−^^3^
0 constraints	

### Fractional atomic coordinates and isotropic or equivalent isotropic displacement parameters (Å^2^)

**Table:**

	*x*	*y*	*z*	*U*_iso_*/*U*_eq_	Occ. (<1)
C5C	−0.0868 (7)	0.8339 (10)	0.7819 (3)	0.101 (3)	0.797 (11)
H7	−0.1013	0.7976	0.8056	0.152*	0.797 (11)
H8	−0.0665	0.7659	0.7626	0.152*	0.797 (11)
H9	−0.1666	0.8673	0.7722	0.152*	0.797 (11)
S3	0.0486 (3)	0.9635 (4)	0.79199 (5)	0.0627 (8)	0.797 (11)
C1	−0.074 (3)	0.738 (2)	0.7612 (9)	0.108 (6)	0.203 (11)
H16	−0.1206	0.6849	0.7777	0.162*	0.203 (11)
H17	−0.0078	0.6911	0.7499	0.162*	0.203 (11)
H18	−0.1374	0.7570	0.7407	0.162*	0.203 (11)
S3'	0.0055 (12)	0.8874 (18)	0.7892 (2)	0.072 (3)	0.203 (11)
C1A	0.3058 (3)	0.6338 (3)	0.48764 (9)	0.0393 (7)	
C1B	−0.4152 (3)	0.5374 (3)	0.34840 (9)	0.0408 (7)	
C2A	0.4370 (3)	0.6700 (3)	0.46797 (9)	0.0408 (7)	
C2B	−0.5618 (4)	0.4670 (4)	0.34575 (10)	0.0482 (8)	
C3A	0.5131 (3)	0.7805 (3)	0.49689 (9)	0.0403 (7)	
C3B	−0.6424 (4)	0.5603 (4)	0.32766 (11)	0.0558 (9)	
C4A	0.4468 (3)	0.8042 (3)	0.52945 (9)	0.0369 (7)	
C4B	−0.5613 (4)	0.6593 (4)	0.31486 (10)	0.0542 (9)	
C5A	0.4410 (4)	0.8588 (4)	0.60993 (11)	0.0636 (11)	
H1	0.4460	0.7655	0.6075	0.095*	
H2	0.3488	0.8733	0.6099	0.095*	
H3	0.4923	0.9027	0.6341	0.095*	
C5B	−0.540 (7)	0.893 (5)	0.282 (2)	0.106 (6)	0.15 (2)
H4	−0.4535	0.8672	0.2771	0.159*	0.15 (2)
H5	−0.5284	0.9640	0.3030	0.159*	0.15 (2)
H6	−0.5805	0.9207	0.2584	0.159*	0.15 (2)
C2	−0.4941 (10)	0.8501 (10)	0.2651 (3)	0.094 (3)	0.85 (2)
H19	−0.4300	0.9119	0.2831	0.142*	0.85 (2)
H20	−0.5329	0.8950	0.2451	0.142*	0.85 (2)
H21	−0.4497	0.7804	0.2533	0.142*	0.85 (2)
C6A	0.3863 (4)	0.7211 (4)	0.43000 (10)	0.0467 (8)	
C6B	−0.5640 (4)	0.3353 (4)	0.31823 (11)	0.0527 (9)	
C7A	0.2434 (4)	0.7296 (4)	0.43012 (10)	0.0520 (9)	
C7B	−0.4258 (4)	0.3248 (3)	0.31042 (10)	0.0469 (8)	
C8A	0.1947 (3)	0.6753 (3)	0.46124 (9)	0.0444 (8)	
C8B	−0.3394 (3)	0.4343 (3)	0.32883 (9)	0.0409 (7)	
C9A	0.0578 (4)	0.6616 (4)	0.46453 (12)	0.0557 (9)	
H9A	0.0234	0.6243	0.4851	0.067*	
C9B	−0.2021 (4)	0.4392 (4)	0.32877 (10)	0.0510 (9)	
H9B	−0.1431	0.5130	0.3413	0.061*	
C10A	−0.0254 (4)	0.7053 (5)	0.43626 (14)	0.0724 (13)	
H10A	−0.1175	0.6969	0.4378	0.087*	
C10B	−0.1548 (5)	0.3312 (4)	0.30955 (12)	0.0639 (11)	
H10B	−0.0625	0.3312	0.3097	0.077*	
C11A	0.0245 (5)	0.7610 (5)	0.40576 (14)	0.0820 (15)	
H11A	−0.0344	0.7901	0.3873	0.098*	
C11B	−0.2432 (5)	0.2229 (4)	0.29000 (13)	0.0687 (12)	
H11B	−0.2092	0.1523	0.2766	0.082*	
C12A	0.1586 (5)	0.7740 (5)	0.40212 (12)	0.0701 (12)	
H12A	0.1922	0.8116	0.3815	0.084*	
C12B	−0.3785 (5)	0.2177 (4)	0.29002 (11)	0.0597 (10)	
H12B	−0.4376	0.1450	0.2768	0.072*	
C13A	0.2158 (3)	0.7433 (3)	0.54657 (9)	0.0383 (7)	
C13B	−0.3150 (4)	0.7404 (3)	0.32166 (10)	0.0472 (8)	
C14A	0.1567 (3)	0.8551 (3)	0.54164 (10)	0.0438 (8)	
H14A	0.1977	0.9204	0.5289	0.053*	
C14B	−0.2340 (5)	0.7157 (4)	0.29249 (12)	0.0696 (12)	
H14B	−0.2553	0.6394	0.2741	0.084*	
C15B	−0.1219 (5)	0.8049 (5)	0.29082 (15)	0.0800 (14)	
H15B	−0.0675	0.7865	0.2713	0.096*	
C16A	−0.0278 (3)	0.7719 (4)	0.57473 (10)	0.0498 (8)	
C16B	−0.0875 (4)	0.9188 (4)	0.31660 (13)	0.0625 (11)	
C15A	0.0347 (4)	0.8687 (4)	0.55607 (11)	0.0498 (8)	
H15A	−0.0053	0.9442	0.5531	0.060*	
C17A	0.0366 (4)	0.6634 (4)	0.58047 (11)	0.0524 (9)	
H17A	−0.0022	0.5996	0.5941	0.063*	
C18A	0.1573 (3)	0.6482 (4)	0.56627 (10)	0.0468 (8)	
H18A	0.1988	0.5742	0.5700	0.056*	
C17B	−0.1718 (4)	0.9422 (4)	0.34562 (11)	0.0576 (10)	
H17B	−0.1522	1.0200	0.3634	0.069*	
C18B	−0.2816 (4)	0.8545 (4)	0.34848 (10)	0.0496 (8)	
H18B	−0.3342	0.8714	0.3686	0.060*	
C19A	−0.1661 (4)	0.7826 (5)	0.58720 (14)	0.0724 (12)	
H19A	−0.2325	0.7221	0.5695	0.109*	
H19B	−0.1857	0.8715	0.5868	0.109*	
H19C	−0.1679	0.7608	0.6133	0.109*	
C19B	0.0391 (5)	1.0120 (6)	0.31561 (17)	0.0941 (17)	
H19D	0.0805	0.9835	0.2928	0.141*	
H19E	0.1004	1.0120	0.3388	0.141*	
H19F	0.0173	1.0999	0.3146	0.141*	
N1A	0.3318 (2)	0.7197 (3)	0.52688 (7)	0.0366 (6)	
N1B	−0.4299 (3)	0.6477 (3)	0.32421 (8)	0.0446 (7)	
N2A	0.6351 (3)	0.8495 (3)	0.49127 (9)	0.0523 (7)	
N2B	−0.7813 (4)	0.5448 (5)	0.32615 (11)	0.0795 (12)	
O1A	0.4557 (3)	0.7450 (3)	0.40438 (7)	0.0596 (7)	
O1B	−0.6620 (3)	0.2552 (3)	0.30708 (10)	0.0868 (10)	
O2A	0.2734 (2)	0.5022 (2)	0.49147 (7)	0.0484 (6)	
H2A	0.3352	0.4785	0.5049	0.073*	
O3B	−0.5922 (3)	0.4394 (3)	0.38327 (7)	0.0563 (7)	
H3B	−0.6712	0.4060	0.3816	0.084*	
O3A	0.5110 (3)	0.5622 (3)	0.46113 (7)	0.0504 (6)	
H3A	0.4885	0.5238	0.4387	0.076*	
O2B	−0.3484 (2)	0.5870 (2)	0.38529 (6)	0.0448 (5)	
H2B	−0.3891	0.6430	0.3960	0.067*	
O4A	0.6824 (3)	0.9511 (3)	0.51445 (9)	0.0707 (8)	
O4B	−0.8460 (3)	0.6210 (5)	0.30993 (11)	0.1101 (14)	
O5A	0.6916 (3)	0.8092 (3)	0.46307 (9)	0.0767 (9)	
O5B	−0.8371 (3)	0.4541 (5)	0.34225 (12)	0.0987 (12)	
S1	0.50842 (9)	0.92446 (9)	0.56943 (3)	0.0504 (2)	
S2	−0.62570 (16)	0.78016 (15)	0.29113 (4)	0.0902 (4)	
C1D	0.6383 (3)	0.8124 (3)	1.01223 (9)	0.0405 (7)	
C2D	0.6902 (3)	0.9583 (3)	1.03256 (9)	0.0422 (7)	
C3D	0.7771 (3)	1.0098 (3)	1.00404 (9)	0.0417 (7)	
C4D	0.7760 (3)	0.9169 (3)	0.97153 (9)	0.0379 (7)	
C5D	0.7641 (5)	0.8362 (5)	0.89118 (11)	0.0664 (11)	
H10	0.6699	0.8350	0.8935	0.100*	
H11	0.7871	0.7483	0.8912	0.100*	
H12	0.7833	0.8668	0.8671	0.100*	
C6D	0.7756 (3)	0.9422 (4)	1.07046 (10)	0.0475 (8)	
C7D	0.7847 (4)	0.8024 (4)	1.07031 (10)	0.0513 (9)	
C8D	0.7035 (3)	0.7262 (4)	1.03883 (10)	0.0451 (8)	
C9D	0.6886 (4)	0.5892 (4)	1.03508 (12)	0.0578 (10)	
H9D	0.6333	0.5371	1.0141	0.069*	
C10D	0.7581 (5)	0.5323 (5)	1.06343 (15)	0.0710 (13)	
H10D	0.7495	0.4405	1.0614	0.085*	
C11D	0.8397 (5)	0.6084 (5)	1.09461 (15)	0.0810 (15)	
H11D	0.8860	0.5671	1.1131	0.097*	
C12D	0.8538 (5)	0.7432 (5)	1.09896 (12)	0.0701 (12)	
H12D	0.9078	0.7944	1.1203	0.084*	
C13D	0.7059 (3)	0.6746 (3)	0.95415 (9)	0.0404 (7)	
C14D	0.8240 (3)	0.6224 (3)	0.95949 (10)	0.0461 (8)	
H14D	0.9012	0.6754	0.9726	0.055*	
C15D	0.8284 (4)	0.4909 (4)	0.94538 (11)	0.0512 (9)	
H15D	0.9088	0.4560	0.9493	0.061*	
C16D	0.7156 (4)	0.4109 (4)	0.92571 (11)	0.0533 (9)	
C17D	0.5980 (4)	0.4670 (4)	0.91898 (11)	0.0539 (9)	
H17D	0.5220	0.4152	0.9047	0.065*	
C18D	0.5921 (4)	0.5971 (4)	0.93292 (10)	0.0477 (8)	
H18D	0.5128	0.6331	0.9282	0.057*	
C19D	0.7187 (5)	0.2650 (4)	0.91262 (15)	0.0778 (13)	
H19J	0.6730	0.2137	0.9298	0.117*	
H19K	0.8104	0.2483	0.9135	0.117*	
H19L	0.6744	0.2404	0.8863	0.117*	
N1D	0.6938 (3)	0.8045 (3)	0.97352 (7)	0.0379 (6)	
N2D	0.8481 (3)	1.1362 (3)	1.00990 (9)	0.0531 (7)	
O1D	0.8237 (3)	1.0340 (3)	1.09637 (7)	0.0595 (7)	
O2D	0.5001 (2)	0.7761 (3)	1.00695 (8)	0.0506 (6)	
H2D	0.4646	0.8271	0.9942	0.076*	
O3D	0.5852 (2)	1.0344 (3)	1.03922 (7)	0.0498 (6)	
H3D	0.5644	1.0295	1.0613	0.075*	
O4D	0.9295 (3)	1.1659 (3)	0.98715 (9)	0.0699 (8)	
O5D	0.8301 (3)	1.2162 (3)	1.03853 (9)	0.0744 (8)	
S4	0.86152 (10)	0.94568 (10)	0.93187 (3)	0.0532 (2)	
C1C	0.3302 (3)	0.7827 (3)	0.84914 (9)	0.0427 (7)	
C2C	0.4071 (4)	0.9207 (4)	0.84560 (10)	0.0465 (8)	
C3C	0.3014 (4)	0.9892 (4)	0.82676 (10)	0.0531 (9)	
C4C	0.1834 (4)	0.9043 (4)	0.81611 (10)	0.0535 (9)	
C6C	0.5135 (4)	0.8886 (4)	0.81767 (10)	0.0505 (9)	
C7C	0.5025 (3)	0.7446 (4)	0.80862 (9)	0.0457 (8)	
C8C	0.4066 (3)	0.6835 (3)	0.82799 (9)	0.0436 (8)	
C9C	0.3899 (4)	0.5475 (4)	0.82721 (11)	0.0593 (10)	
H9C	0.3254	0.5054	0.8404	0.071*	
C10C	0.4731 (5)	0.4761 (5)	0.80591 (13)	0.0739 (12)	
H10C	0.4655	0.3847	0.8054	0.089*	
C11C	0.5665 (5)	0.5370 (5)	0.78551 (13)	0.0716 (12)	
H11C	0.6191	0.4863	0.7709	0.086*	
C12C	0.5829 (4)	0.6709 (5)	0.78655 (11)	0.0595 (10)	
H12C	0.6462	0.7124	0.7728	0.071*	
C13C	0.0970 (3)	0.6701 (4)	0.82203 (10)	0.0499 (9)	
C14C	0.0850 (5)	0.5777 (6)	0.78867 (13)	0.0871 (16)	
H14C	0.1385	0.5917	0.7689	0.105*	
C15C	−0.0070 (6)	0.4646 (6)	0.78502 (16)	0.0958 (18)	
H15C	−0.0155	0.4032	0.7624	0.115*	
C16C	−0.0872 (5)	0.4396 (5)	0.81398 (15)	0.0755 (13)	
C17C	−0.0760 (4)	0.5352 (4)	0.84590 (12)	0.0609 (10)	
H17C	−0.1322	0.5235	0.8651	0.073*	
C18C	0.0158 (4)	0.6483 (4)	0.85049 (11)	0.0529 (9)	
H18C	0.0228	0.7102	0.8730	0.063*	
C19C	−0.1815 (6)	0.3122 (6)	0.8111 (2)	0.116 (2)	
H19G	−0.1368	0.2500	0.8242	0.174*	
H19H	−0.2078	0.2761	0.7841	0.174*	
H19I	−0.2599	0.3293	0.8233	0.174*	
N1C	0.1946 (3)	0.7845 (3)	0.82644 (8)	0.0491 (7)	
N2C	0.3258 (4)	1.1234 (4)	0.82451 (11)	0.0724 (10)	
O1C	0.5932 (3)	0.9711 (3)	0.80765 (10)	0.0791 (9)	
O2C	0.3173 (2)	0.7522 (2)	0.88630 (6)	0.0471 (6)	
H2C	0.2759	0.8057	0.8979	0.071*	
O3C	0.4712 (3)	0.9862 (3)	0.88256 (7)	0.0550 (6)	
H3C	0.5082	1.0599	0.8801	0.083*	
O4C	0.2372 (4)	1.1761 (4)	0.80671 (12)	0.1060 (13)	
O5C	0.4335 (4)	1.1883 (3)	0.84059 (12)	0.0951 (11)	

### Atomic displacement parameters (Å^2^)

**Table:**

	*U*^11^	*U*^22^	*U*^33^	*U*^12^	*U*^13^	*U*^23^
C5C	0.057 (4)	0.118 (7)	0.130 (7)	−0.005 (5)	−0.020 (4)	0.070 (6)
S3	0.0530 (11)	0.075 (2)	0.0635 (9)	0.0199 (12)	−0.0072 (7)	0.0244 (9)
C1	0.070 (9)	0.125 (12)	0.131 (12)	0.003 (10)	−0.026 (9)	0.063 (10)
S3'	0.061 (5)	0.084 (9)	0.071 (4)	0.014 (6)	−0.021 (3)	0.029 (4)
C1A	0.0416 (17)	0.0419 (19)	0.0355 (16)	0.0149 (14)	−0.0010 (13)	0.0056 (13)
C1B	0.0479 (18)	0.0448 (19)	0.0313 (16)	0.0139 (14)	0.0000 (13)	0.0085 (13)
C2A	0.0441 (18)	0.049 (2)	0.0339 (16)	0.0247 (15)	0.0046 (13)	0.0079 (14)
C2B	0.0484 (19)	0.061 (2)	0.0351 (17)	0.0101 (16)	0.0030 (14)	0.0077 (15)
C3A	0.0396 (17)	0.0465 (19)	0.0388 (17)	0.0160 (14)	0.0046 (13)	0.0115 (14)
C3B	0.047 (2)	0.076 (3)	0.046 (2)	0.0205 (19)	−0.0019 (16)	0.0096 (18)
C4A	0.0402 (17)	0.0377 (17)	0.0368 (16)	0.0163 (13)	0.0035 (13)	0.0107 (13)
C4B	0.069 (2)	0.064 (2)	0.0310 (17)	0.026 (2)	−0.0041 (16)	0.0033 (16)
C5A	0.076 (3)	0.076 (3)	0.0365 (19)	0.015 (2)	0.0023 (18)	0.0005 (18)
C5B	0.156 (11)	0.073 (10)	0.080 (11)	0.032 (8)	−0.044 (9)	0.009 (9)
C2	0.147 (5)	0.059 (5)	0.070 (5)	0.007 (4)	−0.034 (3)	0.028 (4)
C6A	0.060 (2)	0.050 (2)	0.0351 (17)	0.0277 (17)	0.0061 (16)	0.0086 (15)
C6B	0.052 (2)	0.059 (2)	0.0442 (19)	0.0011 (18)	−0.0030 (16)	0.0087 (16)
C7A	0.061 (2)	0.058 (2)	0.0409 (19)	0.0327 (18)	−0.0027 (16)	0.0042 (16)
C7B	0.060 (2)	0.045 (2)	0.0383 (17)	0.0141 (16)	0.0007 (15)	0.0113 (15)
C8A	0.0475 (19)	0.046 (2)	0.0382 (17)	0.0197 (15)	−0.0056 (14)	−0.0029 (14)
C8B	0.0508 (19)	0.0456 (19)	0.0291 (15)	0.0136 (15)	0.0019 (13)	0.0114 (13)
C9A	0.050 (2)	0.059 (2)	0.055 (2)	0.0192 (17)	−0.0079 (17)	−0.0032 (17)
C9B	0.056 (2)	0.057 (2)	0.0424 (19)	0.0153 (17)	0.0079 (16)	0.0082 (16)
C10A	0.053 (2)	0.076 (3)	0.082 (3)	0.031 (2)	−0.020 (2)	−0.012 (2)
C10B	0.071 (3)	0.073 (3)	0.054 (2)	0.031 (2)	0.013 (2)	0.008 (2)
C11A	0.087 (3)	0.100 (4)	0.064 (3)	0.057 (3)	−0.018 (2)	0.012 (3)
C11B	0.093 (3)	0.061 (3)	0.060 (3)	0.038 (2)	0.015 (2)	0.010 (2)
C12A	0.085 (3)	0.083 (3)	0.051 (2)	0.046 (2)	−0.005 (2)	0.018 (2)
C12B	0.083 (3)	0.047 (2)	0.048 (2)	0.014 (2)	0.0016 (19)	0.0047 (17)
C13A	0.0371 (16)	0.0443 (18)	0.0353 (16)	0.0112 (14)	0.0052 (13)	0.0061 (13)
C13B	0.064 (2)	0.0405 (19)	0.0389 (18)	0.0115 (16)	0.0007 (16)	0.0121 (14)
C14A	0.0467 (19)	0.0424 (19)	0.0449 (18)	0.0088 (15)	0.0101 (15)	0.0099 (14)
C14B	0.109 (4)	0.048 (2)	0.054 (2)	0.007 (2)	0.029 (2)	0.0027 (18)
C15B	0.101 (4)	0.071 (3)	0.082 (3)	0.018 (3)	0.044 (3)	0.031 (3)
C16A	0.0438 (19)	0.063 (2)	0.0443 (19)	0.0110 (17)	0.0106 (15)	0.0050 (16)
C16B	0.069 (3)	0.058 (3)	0.064 (3)	0.010 (2)	0.001 (2)	0.028 (2)
C15A	0.048 (2)	0.051 (2)	0.054 (2)	0.0198 (16)	0.0075 (16)	0.0055 (16)
C17A	0.052 (2)	0.058 (2)	0.050 (2)	0.0025 (17)	0.0115 (16)	0.0175 (17)
C18A	0.0486 (19)	0.047 (2)	0.0482 (19)	0.0121 (15)	0.0079 (16)	0.0146 (15)
C17B	0.070 (3)	0.050 (2)	0.048 (2)	0.0104 (19)	−0.0110 (19)	0.0051 (17)
C18B	0.062 (2)	0.051 (2)	0.0365 (18)	0.0173 (18)	−0.0005 (16)	0.0034 (15)
C19A	0.050 (2)	0.095 (3)	0.075 (3)	0.015 (2)	0.020 (2)	0.009 (2)
C19B	0.085 (3)	0.103 (4)	0.098 (4)	0.000 (3)	0.003 (3)	0.048 (3)
N1A	0.0367 (14)	0.0413 (15)	0.0333 (13)	0.0112 (11)	0.0042 (11)	0.0051 (11)
N1B	0.0565 (17)	0.0438 (16)	0.0341 (14)	0.0151 (13)	−0.0032 (12)	0.0069 (12)
N2A	0.0452 (17)	0.064 (2)	0.0539 (18)	0.0153 (15)	0.0097 (15)	0.0206 (16)
N2B	0.052 (2)	0.126 (4)	0.062 (2)	0.028 (2)	−0.0060 (18)	0.015 (2)
O1A	0.0809 (18)	0.0702 (18)	0.0390 (13)	0.0384 (14)	0.0152 (13)	0.0165 (12)
O1B	0.069 (2)	0.088 (2)	0.089 (2)	−0.0166 (18)	0.0012 (17)	−0.0097 (18)
O2A	0.0506 (14)	0.0431 (14)	0.0499 (14)	0.0124 (11)	−0.0046 (11)	0.0044 (10)
O3B	0.0530 (14)	0.0763 (18)	0.0403 (13)	0.0035 (13)	0.0076 (11)	0.0151 (12)
O3A	0.0618 (15)	0.0618 (16)	0.0353 (12)	0.0368 (13)	0.0058 (11)	0.0083 (11)
O2B	0.0478 (13)	0.0540 (14)	0.0326 (11)	0.0169 (11)	−0.0012 (9)	0.0018 (10)
O4A	0.0630 (18)	0.078 (2)	0.0673 (18)	−0.0103 (15)	0.0089 (14)	0.0168 (16)
O4B	0.071 (2)	0.181 (4)	0.091 (3)	0.062 (2)	−0.0104 (19)	0.040 (3)
O5A	0.0607 (17)	0.100 (2)	0.079 (2)	0.0227 (16)	0.0366 (16)	0.0176 (17)
O5B	0.0479 (18)	0.145 (4)	0.106 (3)	0.007 (2)	0.0063 (18)	0.036 (3)
S1	0.0530 (5)	0.0503 (5)	0.0445 (5)	0.0062 (4)	−0.0008 (4)	0.0011 (4)
S2	0.1209 (11)	0.0944 (10)	0.0656 (7)	0.0605 (8)	−0.0119 (7)	0.0250 (7)
C1D	0.0443 (18)	0.0436 (19)	0.0372 (17)	0.0137 (14)	0.0080 (14)	0.0093 (13)
C2D	0.0489 (19)	0.047 (2)	0.0342 (16)	0.0232 (15)	0.0019 (14)	0.0062 (14)
C3D	0.0431 (18)	0.0421 (19)	0.0405 (17)	0.0116 (14)	−0.0010 (14)	0.0083 (14)
C4D	0.0376 (16)	0.0435 (18)	0.0362 (16)	0.0175 (14)	0.0035 (13)	0.0093 (13)
C5D	0.084 (3)	0.085 (3)	0.0343 (19)	0.026 (2)	0.0094 (19)	0.0071 (19)
C6D	0.0464 (19)	0.066 (2)	0.0342 (17)	0.0251 (17)	0.0038 (14)	0.0061 (16)
C7D	0.055 (2)	0.067 (2)	0.0415 (19)	0.0314 (18)	0.0075 (16)	0.0180 (17)
C8D	0.0483 (19)	0.053 (2)	0.0415 (18)	0.0206 (16)	0.0113 (15)	0.0167 (15)
C9D	0.065 (2)	0.054 (2)	0.065 (2)	0.0217 (18)	0.0222 (19)	0.0232 (19)
C10D	0.082 (3)	0.064 (3)	0.086 (3)	0.038 (2)	0.030 (3)	0.040 (2)
C11D	0.089 (3)	0.094 (4)	0.080 (3)	0.053 (3)	0.013 (3)	0.046 (3)
C12D	0.074 (3)	0.095 (4)	0.052 (2)	0.045 (2)	0.000 (2)	0.022 (2)
C13D	0.0440 (18)	0.0398 (18)	0.0384 (17)	0.0104 (14)	0.0043 (14)	0.0052 (13)
C14D	0.0437 (18)	0.045 (2)	0.0478 (19)	0.0084 (15)	0.0036 (15)	0.0016 (15)
C15D	0.051 (2)	0.052 (2)	0.053 (2)	0.0197 (17)	0.0053 (16)	0.0043 (16)
C16D	0.066 (2)	0.044 (2)	0.050 (2)	0.0116 (18)	0.0083 (18)	0.0019 (16)
C17D	0.054 (2)	0.050 (2)	0.052 (2)	0.0018 (17)	−0.0026 (17)	0.0001 (17)
C18D	0.0448 (19)	0.049 (2)	0.049 (2)	0.0120 (15)	−0.0014 (15)	0.0043 (16)
C19D	0.096 (3)	0.048 (2)	0.085 (3)	0.014 (2)	0.000 (3)	−0.002 (2)
N1D	0.0417 (14)	0.0375 (15)	0.0352 (14)	0.0085 (11)	0.0055 (11)	0.0041 (11)
N2D	0.0568 (19)	0.0457 (18)	0.0555 (19)	0.0094 (14)	−0.0030 (15)	0.0089 (15)
O1D	0.0647 (16)	0.0750 (19)	0.0386 (13)	0.0288 (14)	−0.0048 (12)	−0.0017 (13)
O2D	0.0445 (13)	0.0559 (16)	0.0567 (15)	0.0154 (11)	0.0105 (11)	0.0164 (12)
O3D	0.0569 (14)	0.0604 (16)	0.0372 (12)	0.0334 (12)	0.0022 (11)	0.0046 (11)
O4D	0.0740 (19)	0.0618 (18)	0.0677 (18)	−0.0120 (15)	0.0015 (15)	0.0127 (14)
O5D	0.096 (2)	0.0473 (16)	0.074 (2)	0.0147 (15)	0.0043 (17)	−0.0111 (14)
S4	0.0569 (5)	0.0618 (6)	0.0452 (5)	0.0105 (4)	0.0132 (4)	0.0156 (4)
C1C	0.0469 (18)	0.053 (2)	0.0286 (15)	0.0117 (15)	−0.0013 (13)	0.0077 (14)
C2C	0.055 (2)	0.048 (2)	0.0377 (17)	0.0136 (16)	−0.0001 (15)	0.0109 (14)
C3C	0.065 (2)	0.054 (2)	0.0433 (19)	0.0186 (19)	0.0000 (17)	0.0135 (16)
C4C	0.062 (2)	0.071 (3)	0.0363 (18)	0.033 (2)	0.0064 (16)	0.0172 (17)
C6C	0.053 (2)	0.060 (2)	0.0411 (19)	0.0109 (18)	0.0034 (16)	0.0167 (16)
C7C	0.0479 (19)	0.056 (2)	0.0331 (16)	0.0120 (16)	−0.0023 (14)	0.0062 (14)
C8C	0.0475 (19)	0.047 (2)	0.0354 (17)	0.0100 (15)	−0.0030 (14)	0.0053 (14)
C9C	0.075 (3)	0.056 (2)	0.046 (2)	0.010 (2)	0.0071 (19)	0.0058 (17)
C10C	0.102 (4)	0.061 (3)	0.061 (3)	0.025 (2)	0.011 (3)	0.004 (2)
C11C	0.086 (3)	0.075 (3)	0.057 (2)	0.039 (2)	0.011 (2)	−0.003 (2)
C12C	0.057 (2)	0.078 (3)	0.045 (2)	0.019 (2)	0.0059 (17)	0.0065 (19)
C13C	0.0452 (19)	0.070 (2)	0.0357 (18)	0.0179 (17)	−0.0005 (15)	0.0069 (16)
C14C	0.067 (3)	0.130 (5)	0.051 (3)	−0.007 (3)	0.009 (2)	−0.017 (3)
C15C	0.086 (4)	0.106 (4)	0.075 (3)	−0.006 (3)	−0.002 (3)	−0.033 (3)
C16C	0.060 (3)	0.077 (3)	0.081 (3)	0.002 (2)	−0.018 (2)	0.009 (3)
C17C	0.054 (2)	0.080 (3)	0.052 (2)	0.010 (2)	0.0001 (18)	0.026 (2)
C18C	0.055 (2)	0.063 (2)	0.0428 (19)	0.0152 (18)	0.0033 (16)	0.0103 (17)
C19C	0.105 (4)	0.089 (4)	0.136 (6)	−0.017 (3)	−0.033 (4)	0.019 (4)
N1C	0.0429 (16)	0.067 (2)	0.0382 (15)	0.0101 (14)	0.0003 (12)	0.0143 (14)
N2C	0.093 (3)	0.061 (2)	0.071 (2)	0.029 (2)	0.004 (2)	0.0287 (19)
O1C	0.083 (2)	0.073 (2)	0.088 (2)	0.0013 (17)	0.0293 (18)	0.0309 (17)
O2C	0.0581 (14)	0.0550 (15)	0.0312 (11)	0.0168 (11)	0.0055 (10)	0.0096 (10)
O3C	0.0694 (16)	0.0526 (15)	0.0407 (13)	0.0047 (12)	−0.0028 (12)	0.0089 (11)
O4C	0.131 (3)	0.083 (2)	0.111 (3)	0.041 (2)	−0.018 (2)	0.042 (2)
O5C	0.108 (3)	0.061 (2)	0.111 (3)	−0.0012 (19)	−0.017 (2)	0.0289 (19)

### Geometric parameters (Å, º)

**Table:**

C5C—S3	1.757 (8)	N2A—O4A	1.241 (4)
S3—C4C	1.737 (4)	N2B—O4B	1.233 (5)
C1—S3'	1.755 (10)	N2B—O5B	1.253 (5)
S3'—C4C	1.909 (10)	C1D—O2D	1.384 (4)
C1A—O2A	1.379 (4)	C1D—N1D	1.503 (4)
C1A—N1A	1.500 (4)	C1D—C8D	1.516 (4)
C1A—C8A	1.509 (4)	C1D—C2D	1.571 (5)
C1A—C2A	1.578 (5)	C2D—O3D	1.412 (4)
C1B—O2B	1.382 (4)	C2D—C3D	1.494 (5)
C1B—N1B	1.508 (4)	C2D—C6D	1.533 (4)
C1B—C8B	1.513 (5)	C3D—C4D	1.376 (5)
C1B—C2B	1.551 (5)	C3D—N2D	1.382 (5)
C2A—O3A	1.419 (4)	C4D—N1D	1.349 (4)
C2A—C3A	1.485 (5)	C4D—S4	1.736 (3)
C2A—C6A	1.541 (4)	C5D—S4	1.800 (4)
C2B—O3B	1.423 (4)	C6D—O1D	1.226 (4)
C2B—C3B	1.489 (5)	C6D—C7D	1.451 (5)
C2B—C6B	1.547 (5)	C7D—C8D	1.379 (5)
C3A—C4A	1.381 (4)	C7D—C12D	1.400 (5)
C3A—N2A	1.387 (4)	C8D—C9D	1.384 (5)
C3B—C4B	1.375 (6)	C9D—C10D	1.381 (6)
C3B—N2B	1.384 (5)	C10D—C11D	1.374 (7)
C4A—N1A	1.348 (4)	C11D—C12D	1.361 (7)
C4A—S1	1.735 (3)	C13D—C14D	1.371 (5)
C4B—N1B	1.354 (5)	C13D—C18D	1.391 (5)
C4B—S2	1.731 (4)	C13D—N1D	1.439 (4)
C5A—S1	1.795 (4)	C14D—C15D	1.383 (5)
C5B—S2	1.45 (6)	C15D—C16D	1.376 (5)
C2—S2	1.809 (10)	C16D—C17D	1.392 (5)
C6A—O1A	1.218 (4)	C16D—C19D	1.514 (6)
C6A—C7A	1.455 (5)	C17D—C18D	1.372 (5)
C6B—O1B	1.199 (4)	N2D—O4D	1.235 (4)
C6B—C7B	1.462 (5)	N2D—O5D	1.247 (4)
C7A—C8A	1.385 (5)	C1C—O2C	1.380 (4)
C7A—C12A	1.385 (5)	C1C—N1C	1.508 (4)
C7B—C8B	1.372 (5)	C1C—C8C	1.512 (5)
C7B—C12B	1.391 (5)	C1C—C2C	1.556 (5)
C8A—C9A	1.389 (5)	C2C—O3C	1.415 (4)
C8B—C9B	1.379 (5)	C2C—C3C	1.489 (5)
C9A—C10A	1.379 (6)	C2C—C6C	1.556 (5)
C9B—C10B	1.381 (5)	C3C—C4C	1.371 (6)
C10A—C11A	1.378 (7)	C3C—N2C	1.384 (5)
C10B—C11B	1.386 (6)	C4C—N1C	1.341 (5)
C11A—C12A	1.364 (7)	C6C—O1C	1.204 (4)
C11B—C12B	1.359 (6)	C6C—C7C	1.460 (5)
C13A—C18A	1.373 (5)	C7C—C8C	1.370 (5)
C13A—C14A	1.380 (5)	C7C—C12C	1.395 (5)
C13A—N1A	1.445 (4)	C8C—C9C	1.384 (5)
C13B—C18B	1.380 (5)	C9C—C10C	1.389 (6)
C13B—C14B	1.382 (5)	C10C—C11C	1.374 (7)
C13B—N1B	1.424 (5)	C11C—C12C	1.361 (6)
C14A—C15A	1.391 (5)	C13C—C18C	1.370 (5)
C14B—C15B	1.375 (6)	C13C—C14C	1.381 (6)
C15B—C16B	1.360 (7)	C13C—N1C	1.417 (5)
C16A—C15A	1.380 (5)	C14C—C15C	1.377 (7)
C16A—C17A	1.385 (5)	C15C—C16C	1.384 (7)
C16A—C19A	1.515 (5)	C16C—C17C	1.365 (7)
C16B—C17B	1.400 (6)	C16C—C19C	1.504 (7)
C16B—C19B	1.504 (6)	C17C—C18C	1.372 (6)
C17A—C18A	1.378 (5)	N2C—O5C	1.237 (5)
C17B—C18B	1.358 (5)	N2C—O4C	1.251 (5)
N2A—O5A	1.232 (4)		
			
C4C—S3—C5C	107.6 (3)	C4B—S2—C2	106.8 (3)
C1—S3'—C4C	122.1 (12)	O2D—C1D—N1D	111.0 (3)
O2A—C1A—N1A	111.3 (2)	O2D—C1D—C8D	109.7 (3)
O2A—C1A—C8A	109.3 (3)	N1D—C1D—C8D	111.5 (3)
N1A—C1A—C8A	111.8 (3)	O2D—C1D—C2D	115.9 (3)
O2A—C1A—C2A	116.1 (3)	N1D—C1D—C2D	104.0 (2)
N1A—C1A—C2A	103.9 (2)	C8D—C1D—C2D	104.6 (3)
C8A—C1A—C2A	104.3 (3)	O3D—C2D—C3D	112.4 (3)
O2B—C1B—N1B	110.6 (3)	O3D—C2D—C6D	113.1 (3)
O2B—C1B—C8B	109.1 (3)	C3D—C2D—C6D	110.8 (3)
N1B—C1B—C8B	111.4 (3)	O3D—C2D—C1D	113.3 (3)
O2B—C1B—C2B	117.7 (3)	C3D—C2D—C1D	102.1 (3)
N1B—C1B—C2B	103.0 (3)	C6D—C2D—C1D	104.4 (3)
C8B—C1B—C2B	104.9 (3)	C4D—C3D—N2D	124.9 (3)
O3A—C2A—C3A	112.3 (3)	C4D—C3D—C2D	111.7 (3)
O3A—C2A—C6A	113.0 (3)	N2D—C3D—C2D	123.4 (3)
C3A—C2A—C6A	110.6 (3)	N1D—C4D—C3D	111.3 (3)
O3A—C2A—C1A	113.2 (3)	N1D—C4D—S4	124.7 (2)
C3A—C2A—C1A	102.3 (2)	C3D—C4D—S4	123.8 (3)
C6A—C2A—C1A	104.5 (3)	O1D—C6D—C7D	127.3 (3)
O3B—C2B—C3B	115.9 (3)	O1D—C6D—C2D	124.3 (3)
O3B—C2B—C6B	109.5 (3)	C7D—C6D—C2D	108.4 (3)
C3B—C2B—C6B	112.7 (3)	C8D—C7D—C12D	120.9 (4)
O3B—C2B—C1B	110.7 (3)	C8D—C7D—C6D	111.2 (3)
C3B—C2B—C1B	102.9 (3)	C12D—C7D—C6D	127.7 (4)
C6B—C2B—C1B	104.3 (3)	C7D—C8D—C9D	120.4 (3)
C4A—C3A—N2A	125.3 (3)	C7D—C8D—C1D	111.1 (3)
C4A—C3A—C2A	111.3 (3)	C9D—C8D—C1D	128.5 (3)
N2A—C3A—C2A	123.4 (3)	C10D—C9D—C8D	118.0 (4)
C4B—C3B—N2B	127.3 (4)	C11D—C10D—C9D	121.5 (4)
C4B—C3B—C2B	111.5 (3)	C12D—C11D—C10D	121.1 (4)
N2B—C3B—C2B	121.2 (4)	C11D—C12D—C7D	118.1 (4)
N1A—C4A—C3A	111.7 (3)	C14D—C13D—C18D	120.0 (3)
N1A—C4A—S1	124.5 (2)	C14D—C13D—N1D	120.8 (3)
C3A—C4A—S1	123.7 (3)	C18D—C13D—N1D	119.0 (3)
N1B—C4B—C3B	110.3 (3)	C13D—C14D—C15D	120.0 (3)
N1B—C4B—S2	127.2 (3)	C16D—C15D—C14D	120.9 (3)
C3B—C4B—S2	122.4 (3)	C15D—C16D—C17D	118.3 (3)
O1A—C6A—C7A	127.5 (3)	C15D—C16D—C19D	120.7 (4)
O1A—C6A—C2A	124.4 (3)	C17D—C16D—C19D	121.0 (4)
C7A—C6A—C2A	108.0 (3)	C18D—C17D—C16D	121.3 (3)
O1B—C6B—C7B	127.3 (4)	C17D—C18D—C13D	119.3 (3)
O1B—C6B—C2B	125.0 (4)	C4D—N1D—C13D	125.5 (3)
C7B—C6B—C2B	107.6 (3)	C4D—N1D—C1D	110.6 (3)
C8A—C7A—C12A	121.5 (4)	C13D—N1D—C1D	117.1 (2)
C8A—C7A—C6A	110.9 (3)	O4D—N2D—O5D	121.9 (3)
C12A—C7A—C6A	127.4 (4)	O4D—N2D—C3D	119.2 (3)
C8B—C7B—C12B	121.4 (4)	O5D—N2D—C3D	118.9 (3)
C8B—C7B—C6B	110.9 (3)	C4D—S4—C5D	103.93 (19)
C12B—C7B—C6B	127.5 (4)	O2C—C1C—N1C	111.3 (3)
C7A—C8A—C9A	120.1 (3)	O2C—C1C—C8C	109.1 (3)
C7A—C8A—C1A	111.8 (3)	N1C—C1C—C8C	110.9 (3)
C9A—C8A—C1A	128.1 (3)	O2C—C1C—C2C	117.8 (3)
C7B—C8B—C9B	120.6 (3)	N1C—C1C—C2C	102.4 (3)
C7B—C8B—C1B	111.3 (3)	C8C—C1C—C2C	105.0 (3)
C9B—C8B—C1B	128.1 (3)	O3C—C2C—C3C	115.0 (3)
C10A—C9A—C8A	117.7 (4)	O3C—C2C—C6C	110.4 (3)
C8B—C9B—C10B	118.0 (4)	C3C—C2C—C6C	111.7 (3)
C11A—C10A—C9A	121.7 (4)	O3C—C2C—C1C	111.2 (3)
C9B—C10B—C11B	120.8 (4)	C3C—C2C—C1C	103.6 (3)
C12A—C11A—C10A	121.1 (4)	C6C—C2C—C1C	104.2 (3)
C12B—C11B—C10B	121.3 (4)	C4C—C3C—N2C	128.4 (4)
C11A—C12A—C7A	117.9 (4)	C4C—C3C—C2C	110.6 (3)
C11B—C12B—C7B	117.8 (4)	N2C—C3C—C2C	120.7 (4)
C18A—C13A—C14A	120.8 (3)	N1C—C4C—C3C	111.3 (3)
C18A—C13A—N1A	119.8 (3)	N1C—C4C—S3	130.8 (4)
C14A—C13A—N1A	119.1 (3)	C3C—C4C—S3	117.9 (3)
C18B—C13B—C14B	119.1 (4)	N1C—C4C—S3'	104.9 (6)
C18B—C13B—N1B	120.7 (3)	C3C—C4C—S3'	143.7 (6)
C14B—C13B—N1B	120.1 (3)	O1C—C6C—C7C	128.1 (4)
C13A—C14A—C15A	119.0 (3)	O1C—C6C—C2C	124.1 (4)
C15B—C14B—C13B	119.5 (4)	C7C—C6C—C2C	107.7 (3)
C16B—C15B—C14B	122.6 (4)	C8C—C7C—C12C	120.9 (4)
C15A—C16A—C17A	118.5 (3)	C8C—C7C—C6C	111.1 (3)
C15A—C16A—C19A	120.3 (4)	C12C—C7C—C6C	127.8 (4)
C17A—C16A—C19A	121.1 (4)	C7C—C8C—C9C	120.8 (3)
C15B—C16B—C17B	116.8 (4)	C7C—C8C—C1C	111.5 (3)
C15B—C16B—C19B	121.9 (5)	C9C—C8C—C1C	127.7 (3)
C17B—C16B—C19B	121.2 (5)	C8C—C9C—C10C	117.5 (4)
C16A—C15A—C14A	121.0 (3)	C11C—C10C—C9C	121.7 (4)
C18A—C17A—C16A	121.2 (3)	C12C—C11C—C10C	120.6 (4)
C13A—C18A—C17A	119.5 (3)	C11C—C12C—C7C	118.6 (4)
C18B—C17B—C16B	121.9 (4)	C18C—C13C—C14C	119.3 (4)
C17B—C18B—C13B	120.1 (4)	C18C—C13C—N1C	121.3 (3)
C4A—N1A—C13A	126.1 (3)	C14C—C13C—N1C	119.4 (4)
C4A—N1A—C1A	110.4 (2)	C15C—C14C—C13C	119.2 (4)
C13A—N1A—C1A	116.9 (2)	C14C—C15C—C16C	122.1 (5)
C4B—N1B—C13B	127.9 (3)	C17C—C16C—C15C	117.0 (4)
C4B—N1B—C1B	111.0 (3)	C17C—C16C—C19C	120.9 (5)
C13B—N1B—C1B	119.4 (3)	C15C—C16C—C19C	122.1 (5)
O5A—N2A—O4A	122.3 (3)	C16C—C17C—C18C	122.1 (4)
O5A—N2A—C3A	119.0 (3)	C13C—C18C—C17C	120.2 (4)
O4A—N2A—C3A	118.7 (3)	C4C—N1C—C13C	129.8 (3)
O4B—N2B—O5B	122.0 (4)	C4C—N1C—C1C	111.4 (3)
O4B—N2B—C3B	119.8 (5)	C13C—N1C—C1C	118.6 (3)
O5B—N2B—C3B	118.1 (4)	O5C—N2C—O4C	121.8 (4)
C4A—S1—C5A	103.90 (18)	O5C—N2C—C3C	119.3 (4)
C5B—S2—C4B	122 (2)	O4C—N2C—C3C	119.0 (4)
			
O2A—C1A—C2A—O3A	−6.8 (4)	O2D—C1D—C2D—C3D	−126.5 (3)
N1A—C1A—C2A—O3A	115.7 (3)	N1D—C1D—C2D—C3D	−4.4 (3)
C8A—C1A—C2A—O3A	−127.1 (3)	C8D—C1D—C2D—C3D	112.7 (3)
O2A—C1A—C2A—C3A	−127.9 (3)	O2D—C1D—C2D—C6D	118.1 (3)
N1A—C1A—C2A—C3A	−5.5 (3)	N1D—C1D—C2D—C6D	−119.9 (3)
C8A—C1A—C2A—C3A	111.7 (3)	C8D—C1D—C2D—C6D	−2.8 (3)
O2A—C1A—C2A—C6A	116.7 (3)	O3D—C2D—C3D—C4D	−119.6 (3)
N1A—C1A—C2A—C6A	−120.9 (3)	C6D—C2D—C3D—C4D	112.8 (3)
C8A—C1A—C2A—C6A	−3.7 (3)	C1D—C2D—C3D—C4D	2.1 (3)
O2B—C1B—C2B—O3B	−13.3 (4)	O3D—C2D—C3D—N2D	59.7 (4)
N1B—C1B—C2B—O3B	−135.2 (3)	C6D—C2D—C3D—N2D	−67.9 (4)
C8B—C1B—C2B—O3B	108.1 (3)	C1D—C2D—C3D—N2D	−178.6 (3)
O2B—C1B—C2B—C3B	111.2 (3)	N2D—C3D—C4D—N1D	−177.8 (3)
N1B—C1B—C2B—C3B	−10.8 (3)	C2D—C3D—C4D—N1D	1.5 (4)
C8B—C1B—C2B—C3B	−127.4 (3)	N2D—C3D—C4D—S4	−1.5 (5)
O2B—C1B—C2B—C6B	−131.0 (3)	C2D—C3D—C4D—S4	177.8 (2)
N1B—C1B—C2B—C6B	107.1 (3)	O3D—C2D—C6D—O1D	−49.3 (5)
C8B—C1B—C2B—C6B	−9.6 (3)	C3D—C2D—C6D—O1D	77.9 (4)
O3A—C2A—C3A—C4A	−118.8 (3)	C1D—C2D—C6D—O1D	−172.9 (3)
C6A—C2A—C3A—C4A	113.9 (3)	O3D—C2D—C6D—C7D	129.0 (3)
C1A—C2A—C3A—C4A	3.0 (3)	C3D—C2D—C6D—C7D	−103.8 (3)
O3A—C2A—C3A—N2A	61.1 (4)	C1D—C2D—C6D—C7D	5.4 (4)
C6A—C2A—C3A—N2A	−66.2 (4)	O1D—C6D—C7D—C8D	171.8 (4)
C1A—C2A—C3A—N2A	−177.1 (3)	C2D—C6D—C7D—C8D	−6.4 (4)
O3B—C2B—C3B—C4B	130.0 (3)	O1D—C6D—C7D—C12D	−2.4 (6)
C6B—C2B—C3B—C4B	−102.7 (4)	C2D—C6D—C7D—C12D	179.4 (4)
C1B—C2B—C3B—C4B	9.1 (4)	C12D—C7D—C8D—C9D	0.2 (5)
O3B—C2B—C3B—N2B	−48.1 (5)	C6D—C7D—C8D—C9D	−174.4 (3)
C6B—C2B—C3B—N2B	79.1 (4)	C12D—C7D—C8D—C1D	179.2 (3)
C1B—C2B—C3B—N2B	−169.1 (3)	C6D—C7D—C8D—C1D	4.6 (4)
N2A—C3A—C4A—N1A	−178.8 (3)	O2D—C1D—C8D—C7D	−125.8 (3)
C2A—C3A—C4A—N1A	1.1 (4)	N1D—C1D—C8D—C7D	110.8 (3)
N2A—C3A—C4A—S1	−1.1 (5)	C2D—C1D—C8D—C7D	−0.9 (4)
C2A—C3A—C4A—S1	178.8 (2)	O2D—C1D—C8D—C9D	53.1 (5)
N2B—C3B—C4B—N1B	174.8 (4)	N1D—C1D—C8D—C9D	−70.2 (4)
C2B—C3B—C4B—N1B	−3.1 (4)	C2D—C1D—C8D—C9D	178.0 (3)
N2B—C3B—C4B—S2	−4.1 (6)	C7D—C8D—C9D—C10D	−0.6 (5)
C2B—C3B—C4B—S2	177.9 (3)	C1D—C8D—C9D—C10D	−179.5 (3)
O3A—C2A—C6A—O1A	−47.8 (5)	C8D—C9D—C10D—C11D	0.2 (6)
C3A—C2A—C6A—O1A	79.2 (4)	C9D—C10D—C11D—C12D	0.7 (7)
C1A—C2A—C6A—O1A	−171.3 (3)	C10D—C11D—C12D—C7D	−1.2 (7)
O3A—C2A—C6A—C7A	130.0 (3)	C8D—C7D—C12D—C11D	0.7 (6)
C3A—C2A—C6A—C7A	−103.0 (3)	C6D—C7D—C12D—C11D	174.4 (4)
C1A—C2A—C6A—C7A	6.5 (4)	C18D—C13D—C14D—C15D	−3.1 (5)
O3B—C2B—C6B—O1B	65.6 (5)	N1D—C13D—C14D—C15D	170.6 (3)
C3B—C2B—C6B—O1B	−65.0 (5)	C13D—C14D—C15D—C16D	0.4 (6)
C1B—C2B—C6B—O1B	−175.9 (4)	C14D—C15D—C16D—C17D	2.5 (6)
O3B—C2B—C6B—C7B	−111.5 (3)	C14D—C15D—C16D—C19D	−176.1 (4)
C3B—C2B—C6B—C7B	117.9 (3)	C15D—C16D—C17D—C18D	−2.8 (6)
C1B—C2B—C6B—C7B	7.0 (4)	C19D—C16D—C17D—C18D	175.8 (4)
O1A—C6A—C7A—C8A	170.6 (4)	C16D—C17D—C18D—C13D	0.1 (6)
C2A—C6A—C7A—C8A	−7.1 (4)	C14D—C13D—C18D—C17D	2.8 (5)
O1A—C6A—C7A—C12A	−4.1 (7)	N1D—C13D—C18D—C17D	−171.0 (3)
C2A—C6A—C7A—C12A	178.2 (4)	C3D—C4D—N1D—C13D	−154.6 (3)
O1B—C6B—C7B—C8B	−178.4 (4)	S4—C4D—N1D—C13D	29.2 (4)
C2B—C6B—C7B—C8B	−1.4 (4)	C3D—C4D—N1D—C1D	−4.7 (4)
O1B—C6B—C7B—C12B	−3.2 (6)	S4—C4D—N1D—C1D	179.1 (2)
C2B—C6B—C7B—C12B	173.9 (3)	C14D—C13D—N1D—C4D	54.7 (4)
C12A—C7A—C8A—C9A	1.3 (6)	C18D—C13D—N1D—C4D	−131.5 (3)
C6A—C7A—C8A—C9A	−173.7 (3)	C14D—C13D—N1D—C1D	−93.5 (4)
C12A—C7A—C8A—C1A	179.8 (4)	C18D—C13D—N1D—C1D	80.3 (4)
C6A—C7A—C8A—C1A	4.7 (4)	O2D—C1D—N1D—C4D	130.9 (3)
O2A—C1A—C8A—C7A	−125.2 (3)	C8D—C1D—N1D—C4D	−106.5 (3)
N1A—C1A—C8A—C7A	111.2 (3)	C2D—C1D—N1D—C4D	5.7 (3)
C2A—C1A—C8A—C7A	−0.4 (4)	O2D—C1D—N1D—C13D	−76.4 (3)
O2A—C1A—C8A—C9A	53.1 (4)	C8D—C1D—N1D—C13D	46.3 (4)
N1A—C1A—C8A—C9A	−70.6 (4)	C2D—C1D—N1D—C13D	158.4 (3)
C2A—C1A—C8A—C9A	177.9 (3)	C4D—C3D—N2D—O4D	−9.0 (5)
C12B—C7B—C8B—C9B	−2.3 (5)	C2D—C3D—N2D—O4D	171.8 (3)
C6B—C7B—C8B—C9B	173.3 (3)	C4D—C3D—N2D—O5D	172.5 (3)
C12B—C7B—C8B—C1B	179.2 (3)	C2D—C3D—N2D—O5D	−6.7 (5)
C6B—C7B—C8B—C1B	−5.2 (4)	N1D—C4D—S4—C5D	23.7 (3)
O2B—C1B—C8B—C7B	136.3 (3)	C3D—C4D—S4—C5D	−152.1 (3)
N1B—C1B—C8B—C7B	−101.3 (3)	O2C—C1C—C2C—O3C	9.4 (4)
C2B—C1B—C8B—C7B	9.4 (3)	N1C—C1C—C2C—O3C	131.9 (3)
O2B—C1B—C8B—C9B	−42.0 (4)	C8C—C1C—C2C—O3C	−112.2 (3)
N1B—C1B—C8B—C9B	80.4 (4)	O2C—C1C—C2C—C3C	−114.7 (3)
C2B—C1B—C8B—C9B	−168.9 (3)	N1C—C1C—C2C—C3C	7.8 (3)
C7A—C8A—C9A—C10A	−0.8 (5)	C8C—C1C—C2C—C3C	123.7 (3)
C1A—C8A—C9A—C10A	−178.9 (4)	O2C—C1C—C2C—C6C	128.4 (3)
C7B—C8B—C9B—C10B	0.3 (5)	N1C—C1C—C2C—C6C	−109.2 (3)
C1B—C8B—C9B—C10B	178.5 (3)	C8C—C1C—C2C—C6C	6.8 (3)
C8A—C9A—C10A—C11A	−0.2 (6)	O3C—C2C—C3C—C4C	−127.7 (3)
C8B—C9B—C10B—C11B	1.8 (6)	C6C—C2C—C3C—C4C	105.4 (4)
C9A—C10A—C11A—C12A	0.6 (7)	C1C—C2C—C3C—C4C	−6.1 (4)
C9B—C10B—C11B—C12B	−1.9 (6)	O3C—C2C—C3C—N2C	47.7 (5)
C10A—C11A—C12A—C7A	−0.1 (7)	C6C—C2C—C3C—N2C	−79.2 (4)
C8A—C7A—C12A—C11A	−0.9 (6)	C1C—C2C—C3C—N2C	169.3 (3)
C6A—C7A—C12A—C11A	173.3 (4)	N2C—C3C—C4C—N1C	−173.4 (4)
C10B—C11B—C12B—C7B	−0.1 (6)	C2C—C3C—C4C—N1C	1.6 (4)
C8B—C7B—C12B—C11B	2.2 (5)	N2C—C3C—C4C—S3	7.3 (6)
C6B—C7B—C12B—C11B	−172.6 (4)	C2C—C3C—C4C—S3	−177.8 (3)
C18A—C13A—C14A—C15A	−1.7 (5)	N2C—C3C—C4C—S3'	10.8 (9)
N1A—C13A—C14A—C15A	171.2 (3)	C2C—C3C—C4C—S3'	−174.2 (5)
C18B—C13B—C14B—C15B	0.2 (6)	C5C—S3—C4C—N1C	0.5 (5)
N1B—C13B—C14B—C15B	−178.8 (4)	C5C—S3—C4C—C3C	179.7 (4)
C13B—C14B—C15B—C16B	−1.0 (7)	C5C—S3—C4C—S3'	4.6 (7)
C14B—C15B—C16B—C17B	0.3 (7)	C1—S3'—C4C—N1C	−32.4 (16)
C14B—C15B—C16B—C19B	176.9 (5)	C1—S3'—C4C—C3C	143.5 (17)
C17A—C16A—C15A—C14A	3.0 (6)	C1—S3'—C4C—S3	150.8 (18)
C19A—C16A—C15A—C14A	−174.8 (3)	O3C—C2C—C6C—O1C	−60.7 (5)
C13A—C14A—C15A—C16A	−0.6 (5)	C3C—C2C—C6C—O1C	68.7 (5)
C15A—C16A—C17A—C18A	−3.2 (6)	C1C—C2C—C6C—O1C	179.9 (3)
C19A—C16A—C17A—C18A	174.6 (4)	O3C—C2C—C6C—C7C	116.0 (3)
C14A—C13A—C18A—C17A	1.5 (5)	C3C—C2C—C6C—C7C	−114.6 (3)
N1A—C13A—C18A—C17A	−171.4 (3)	C1C—C2C—C6C—C7C	−3.4 (3)
C16A—C17A—C18A—C13A	0.9 (6)	O1C—C6C—C7C—C8C	174.8 (4)
C15B—C16B—C17B—C18B	1.2 (6)	C2C—C6C—C7C—C8C	−1.7 (4)
C19B—C16B—C17B—C18B	−175.5 (4)	O1C—C6C—C7C—C12C	0.5 (6)
C16B—C17B—C18B—C13B	−2.0 (6)	C2C—C6C—C7C—C12C	−176.0 (3)
C14B—C13B—C18B—C17B	1.2 (5)	C12C—C7C—C8C—C9C	2.1 (5)
N1B—C13B—C18B—C17B	−179.8 (3)	C6C—C7C—C8C—C9C	−172.6 (3)
C3A—C4A—N1A—C13A	−155.3 (3)	C12C—C7C—C8C—C1C	−178.8 (3)
S1—C4A—N1A—C13A	27.0 (4)	C6C—C7C—C8C—C1C	6.5 (4)
C3A—C4A—N1A—C1A	−5.1 (3)	O2C—C1C—C8C—C7C	−135.5 (3)
S1—C4A—N1A—C1A	177.2 (2)	N1C—C1C—C8C—C7C	101.5 (3)
C18A—C13A—N1A—C4A	−132.1 (3)	C2C—C1C—C8C—C7C	−8.4 (3)
C14A—C13A—N1A—C4A	54.9 (4)	O2C—C1C—C8C—C9C	43.5 (5)
C18A—C13A—N1A—C1A	79.4 (4)	N1C—C1C—C8C—C9C	−79.5 (4)
C14A—C13A—N1A—C1A	−93.6 (4)	C2C—C1C—C8C—C9C	170.6 (3)
O2A—C1A—N1A—C4A	132.2 (3)	C7C—C8C—C9C—C10C	−0.4 (6)
C8A—C1A—N1A—C4A	−105.3 (3)	C1C—C8C—C9C—C10C	−179.4 (4)
C2A—C1A—N1A—C4A	6.6 (3)	C8C—C9C—C10C—C11C	−1.6 (7)
O2A—C1A—N1A—C13A	−74.6 (3)	C9C—C10C—C11C—C12C	1.9 (7)
C8A—C1A—N1A—C13A	48.0 (4)	C10C—C11C—C12C—C7C	−0.2 (6)
C2A—C1A—N1A—C13A	159.8 (2)	C8C—C7C—C12C—C11C	−1.8 (5)
C3B—C4B—N1B—C13B	−169.6 (3)	C6C—C7C—C12C—C11C	172.0 (4)
S2—C4B—N1B—C13B	9.3 (5)	C18C—C13C—C14C—C15C	−1.0 (7)
C3B—C4B—N1B—C1B	−4.7 (4)	N1C—C13C—C14C—C15C	178.0 (5)
S2—C4B—N1B—C1B	174.2 (3)	C13C—C14C—C15C—C16C	−0.7 (9)
C18B—C13B—N1B—C4B	74.3 (4)	C14C—C15C—C16C—C17C	3.0 (8)
C14B—C13B—N1B—C4B	−106.8 (5)	C14C—C15C—C16C—C19C	−176.1 (6)
C18B—C13B—N1B—C1B	−89.5 (4)	C15C—C16C—C17C—C18C	−3.6 (7)
C14B—C13B—N1B—C1B	89.4 (4)	C19C—C16C—C17C—C18C	175.4 (4)
O2B—C1B—N1B—C4B	−116.6 (3)	C14C—C13C—C18C—C17C	0.4 (6)
C8B—C1B—N1B—C4B	121.9 (3)	N1C—C13C—C18C—C17C	−178.6 (3)
C2B—C1B—N1B—C4B	10.0 (3)	C16C—C17C—C18C—C13C	2.0 (6)
O2B—C1B—N1B—C13B	49.7 (4)	C3C—C4C—N1C—C13C	178.1 (3)
C8B—C1B—N1B—C13B	−71.8 (4)	S3—C4C—N1C—C13C	−2.7 (6)
C2B—C1B—N1B—C13B	176.3 (3)	S3'—C4C—N1C—C13C	−4.5 (5)
C4A—C3A—N2A—O5A	171.8 (3)	C3C—C4C—N1C—C1C	4.1 (4)
C2A—C3A—N2A—O5A	−8.1 (5)	S3—C4C—N1C—C1C	−176.7 (3)
C4A—C3A—N2A—O4A	−9.6 (5)	S3'—C4C—N1C—C1C	−178.5 (3)
C2A—C3A—N2A—O4A	170.5 (3)	C18C—C13C—N1C—C4C	−89.6 (5)
C4B—C3B—N2B—O4B	4.4 (7)	C14C—C13C—N1C—C4C	91.4 (5)
C2B—C3B—N2B—O4B	−177.8 (4)	C18C—C13C—N1C—C1C	84.0 (4)
C4B—C3B—N2B—O5B	−174.2 (4)	C14C—C13C—N1C—C1C	−94.9 (4)
C2B—C3B—N2B—O5B	3.6 (6)	O2C—C1C—N1C—C4C	119.2 (3)
N1A—C4A—S1—C5A	26.9 (3)	C8C—C1C—N1C—C4C	−119.1 (3)
C3A—C4A—S1—C5A	−150.5 (3)	C2C—C1C—N1C—C4C	−7.5 (3)
N1B—C4B—S2—C5B	−6 (4)	O2C—C1C—N1C—C13C	−55.5 (4)
C3B—C4B—S2—C5B	173 (4)	C8C—C1C—N1C—C13C	66.2 (4)
N1B—C4B—S2—C2	21.0 (5)	C2C—C1C—N1C—C13C	177.7 (3)
C3B—C4B—S2—C2	−160.3 (5)	C4C—C3C—N2C—O5C	169.8 (4)
O2D—C1D—C2D—O3D	−5.4 (4)	C2C—C3C—N2C—O5C	−4.7 (6)
N1D—C1D—C2D—O3D	116.7 (3)	C4C—C3C—N2C—O4C	−9.1 (7)
C8D—C1D—C2D—O3D	−126.2 (3)	C2C—C3C—N2C—O4C	176.3 (4)

### Hydrogen-bond geometry (Å, º)

**Table:**

*D*—H···*A*	*D*—H	H···*A*	*D*···*A*	*D*—H···*A*
O3*B*—H3*B*···O5*B*	0.82	2.19	2.748 (4)	125
O3*C*—H3*C*···O5*C*	0.82	2.17	2.728 (4)	126
O2*A*—H2*A*···O3*A*^i^	0.82	1.97	2.785 (3)	175
O3*A*—H3*A*···O3*B*^ii^	0.82	2.04	2.854 (3)	172
O2*B*—H2*B*···O1*A*^iii^	0.82	2.01	2.787 (3)	157
O2*C*—H2*C*···O5*D*^iv^	0.82	2.57	3.133 (4)	127
O2*C*—H2*C*···O1*D*^iv^	0.82	2.03	2.797 (4)	155
O3*D*—H3*D*···O3*C*^iv^	0.82	2.04	2.857 (3)	175
C10*A*—H10*A*···O5*A*^iii^	0.93	2.54	3.347 (5)	146
C10*C*—H10*C*···O5*C*^v^	0.93	2.50	3.331 (6)	149
C10*D*—H10*D*···O5*D*^v^	0.93	2.59	3.437 (5)	151
C10*B*—H10*B*···O5*B*^ii^	0.93	2.53	3.316 (6)	142
C2—H20···O1*C*^vi^	0.96	2.50	3.446 (7)	170

Symmetry codes: (i) −*x*+1, −*y*+1, −*z*+1; (ii) *x*+1, *y*, *z*; (iii) *x*−1, *y*, *z*; (iv) −*x*+1, −*y*+2, −*z*+2; (v) *x*, *y*−1, *z*; (vi) −*x*, −*y*+2, −*z*+1.
